# Wisconsin

**Published:** 1897-04

**Authors:** 


*Wisconsin.* A bill to regulate the practice of veterinary medicine, surgery, and dentistry:

SECTION 1. No person shall practise veterinary medicine, surgery, and dentistry, or any branch thereof, in this State, for compensation, or shall directly or indirectly ask or receive for his service as a practitioner of veterinary medicine and surgery, any fee or reward, or shall assume the title of doctor, physician, or surgeon by means of any abbreviation, or by the use of any word or words, letters of the alphabet of the English or any other language, or any device of whatsoever kind, printed, written, or painted, or exhibited in any advertisement, circular, handbill, letter, or other instrument, nor on any card, sign, door, or place whatsoever; nor shall he be a competent expert witness in any court in a matter pertaining to diseases of animals, except he be duly registered as hereafter provided in the office of the register of deeds of the county in which he resides.

SEC. 2. No person shall be entitled to register as such practitioner unless he be a graduate of a legally chartered school, college, or university of medicine and surgery, or shall have practised veterinary medicine, surgery, and dentistry in this State continuously for a period of not less than five years preceding the passage of this Act; or if he be not a graduate of a legally chartered school, college, or university of medicine, surgery, or dentistry, he must pass an examination before a board of examiners. Should any candidate presenting himself for examination before said board fail to pass the same in a creditable manner in the judgment of said board, he may present an application to said board for another examination at any time within six months after his first examination.

SEC. 3. A board of examiners shall be appointed by the Governor within thirty days after the passage of this Act, to be known as the "State Board of Veterinary Medical Examiners." Such board shall consist of three practising veterinarians, who shall each be the holder of a diploma granted by a legally chartered school, college, or university, who shall hold office one for one year, one for two years, and one for three years after such appointment or until their successors are appointed thereafter each year. The Governor shall appoint one member of said board to fill the vacancy occasioned by the expiration of the term of office of those previously appointed, and is further authorized to fill such vacancies as may occur.

SEC. 4. Provides for a fee of ten dollars, to be used to defray expenses of the board.

SEC. 5. Provides for the proper registration in the respective counties of the State by those who are licensed by the board.

SEC. 6. Provides penalties for violation of the provisions of the Act.

SEC. 7. Designates the county district attorneys as prosecutors.

SEC. 8. Provides for the rendering of gratuitous service in emergency cases by authorized practitioners from other States.

SEC. 9. Provides for the immediate enforcement of the provisions of the Act on passage.

*Connecticut.* The repeal of the former law has led to the presentation of a new one relative to the control of bovine tuberculosis. A bill to regulate the practice of veterinary science has been offered in the present State Legislature.

*Pennsylvania.* The bill to license horseshoers in Pennsylvania was defeated on second reading in the House, and an effort to reconsider it for a place on the calendar was followed by a motion to indefinitely postpone. Public sentiment was not sufficiently aroused as to its merits.

A resolution relating to the protection of the cattle of Pennsylvania from diseased cattle from other States, adopted by the Legislative Committee of the Pennsylvania State Grange, March 9, 1897:

WHEREAS, There are at present no restrictions on the introduction of diseased cattle from other States into Pennsylvania; and

WHEREAS, It is known that many cattle in this Commonwealth have become diseased through contact with diseased cattle from other States; and

WHEREAS, The fact that some States have established regulations providing that only healthy cattle may be brought within their borders places Pennsylvania at a disadvantage in this respect; be it

*Resolved,* That we, the Legislative Committee of Pennsylvania State Grange, after a careful examination of the proposed bill introduced by Hon. Louis Piollet, entitled "An Act to protect the health of the domestic animals of the Commonwealth of Pennsylvania," most heartily indorse the same and earnestly urge its passage by the Legislature.

Committee: Leonard Rhone, S. J. Logan, James G. McSparran, B. F. Warren, Frank N. Moore, and Gerard C. Brown.

---

Dr. Pize has discovered in guaiacol a new anæsthetic. Small doses are injected under the skin.

